# Establishing Correlates of Maternal-Fetal Cytomegalovirus Transmission—One Step Closer Through Predictive Modeling

**DOI:** 10.1093/infdis/jiae281

**Published:** 2024-06-12

**Authors:** Arnaud Marchant, Sancar Adali, Hannah Alsdurf, Vanesa Bol, Xavier Capelle, Nathalie De Schrevel, Jean-Marc Delroisse, Roland Devlieger, Ilse Dieussaert, Catherine Donner, Michel Janssens, Philip Loquet, Anil A Panackal, Claudia Seidl, Robert A van den Berg, Robert Paris

**Affiliations:** European Plotkin Institute for Vaccinology, Université libre de Bruxelles, Brussels, Belgium; GSK, Rockville, Maryland, USA; GSK, Rockville, Maryland, USA; GSK, Rixensart, Belgium; Domaine Universitaire du Sart-Tilman, Service de Gynécologie-Obstétrique, Centre Hospitalier Universitaire de Liège, Belgium; GSK, Rixensart, Belgium; GSK, Wavre, Belgium; Department of Obstetrics and Gynecology, University Hospitals KU Leuven, Belgium; Department of Obstetrics and Gynecology, GZA Ziekenhuizen–Campus Sint-Augustinus, Wilrijk, Belgium; GSK, Rixensart, Belgium; Department of Obstetrics and Gynecology, Hôpital Erasme, Brussels, Belgium; GSK, Rixensart, Belgium; Department of Obstetrics and Gynecology, GZA Ziekenhuizen–Campus Sint-Augustinus, Wilrijk, Belgium; GSK, Rockville, Maryland, USA; GSK, Munich, Germany; GSK, Rockville, Maryland, USA; GSK, Rockville, Maryland, USA

**Keywords:** congenital cytomegalovirus infection, antibodies, viral load, correlate of risk, modeling

## Abstract

**Background:**

Determinants of maternal-fetal cytomegalovirus (CMV) transmission and factors influencing the severity of congenital CMV (cCMV) infection are not well understood.

**Methods:**

We conducted a descriptive, multicenter study in pregnant women ≥18 years old with primary CMV infection and their newborns to explore maternal immune responses to CMV and determine potential immunologic/virologic correlates of cCMV following primary infection during pregnancy. We developed alternative approaches looking into univariate/multivariate factors associated with cCMV, including a participant clustering/stratification approach and an interpretable predictive model–based approach using trained decision trees for risk prediction (post hoc analyses).

**Results:**

Pregnant women were grouped in 3 distinct clusters with similar baseline characteristics, particularly gestational age at diagnosis. We observed a trend for higher viral loads in urine and saliva samples from mothers of infants with cCMV versus without cCMV. When using a trained predictive-model approach that accounts for interaction effects between variables, anti-pentamer immunoglobulin G antibody concentration and viral load in saliva were identified as biomarkers jointly associated with the risk of maternal-fetal CMV transmission.

**Conclusions:**

We identified biomarkers of CMV maternal-fetal transmission. After validation in larger studies, our findings will guide the management of primary infection during pregnancy and the development of vaccines against cCMV.

**Clinical Trials Registration:**

NCT01251744.

## Teaser

Using different models (participant clustering/stratification and an interpretable predictive model using trained decision trees for risk prediction), we found that anti-pentamer IgG concentration during pregnancy and viral load in saliva are biomarkers jointly predicting the risk of maternal-fetal cytomegalovirus transmission.

## Graphical abstract

**Graphical abstract:**
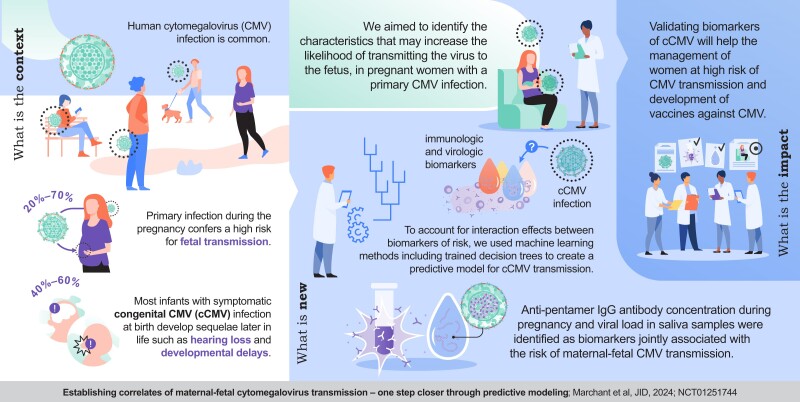

The human cytomegalovirus (CMV) is a ubiquitous herpesvirus, establishing lifelong latency following primary infection [1]. CMV infection can be serious or even life-threatening in patients with deficient or developing immune systems, such as congenitally infected fetuses [1]. CMV is the most common virus causing congenital infection and birth defects; in a recent meta-analysis of data from 36 countries, the pooled overall prevalence of congenital CMV (cCMV) was estimated at 0.67% [2]. Several demographic, clinical, and environmental factors can increase the risk of cCMV [2–5].

While cCMV infection at birth is asymptomatic in around 90% of cases, symptomatic cCMV infection can lead to the development of long-term sequalae in 40%–60% of affected infants, such as sensorineural hearing loss (SNHL), cognitive impairment, retinitis, and cerebral palsy [6]. Even asymptomatic children are at risk, with around 10%–15% showing some developmental disorder, mainly SNHL [6–8]. Primary infection during pregnancy can lead to fetal transmission in 20%–70% of cases [6], and maternal seroconversions occurring in the late first and early second trimesters are associated with more severe neurological outcomes for the infants [7].

While early detection and treatment of cCMV may prevent or decrease the severity of SNHL and improve developmental outcomes in newborns with symptomatic infection [9–11], universal screening at birth is not implemented in most countries. Therefore, establishing predictors of cCMV remains of utmost importance [4]. A biomarker of risk for maternal-fetal CMV transmission would also facilitate the development of a vaccine against cCMV. Several biomarkers have been investigated, including maternal antibodies against the pentameric gH/gL/UL128/UL130/UL131A complex [12–14], CMV-specific immunoglobulin G (IgG) avidity [12, 15], and antibody-dependent cellular phagocytosis [15]. However, the association of clinical and biological factors in CMV-seropositive pregnant women and cCMV infection is poorly investigated. Moreover, the biology and immunology of CMV in pregnant women are not completely elucidated. Therefore, defining a single marker of risk for vertical CMV transmission may not be possible, and the potential interplay of several factors (including clinical and biological) should be explored. In addition, there may exist subsets of mothers with specific clinical and behavioral profiles relevant to cCMV risk. Such risk profiling (eg, through clustering of pregnant infected women with similar characteristics) can be a cost-efficient way of focusing interventions and/or further investigations. We explored these factors and their interaction by using traditional statistical approaches (univariate and multivariate analyses) as well as machine learning methods, including trained decision trees, to create a predictive model for maternal-fetal CMV transmission. We chose to use decision trees due to their ability of handling mixed data types, accommodating missing data, and being more robust to outliers, while also being easily interpretable [16].

In the attempt to identify a correlate of maternal-fetal CMV transmission, we conducted a multicenter study in pregnant women with confirmed primary CMV infection and their offspring to investigate associations between selected clinical, immunologic, and virologic markers and cCMV infection.

## METHODS

### Study Design and Participants

This study was conducted in 10 clinical centers between December 2010 and June 2015 in Belgium and enrolled pregnant women aged 18 years or older with confirmed primary CMV infection and their newborns. CMV screening was routinely proposed to all pregnant women attending the study sites and conducted as per local clinical practice. Inclusion required written informed consent and the participants’ capacity to comply with protocol requirements as assessed by the investigator. Exclusion criteria are detailed in the Supplementary Material.

After study entry, pregnant women were invited to monthly intermediate visits until pregnancy conclusion and to another visit at 1 month post–pregnancy conclusion. All live births were followed up for approximately 10 days, with a 2-year follow-up of the medical status for newborns diagnosed with cCMV infections (Supplementary Figure 1) via chart review and structured interview of the treating physicians to assess any evidence of symptomatic CMV disease. Mothers with confirmed primary CMV infection and whose newborn/fetus had CMV infection status available were included in the analyses and were grouped by their newborn's infection status as follows: 1/multiple newborn(s)/fetus(es) with cCMV (cCMV-positive group), 1/multiple newborn(s)/fetus(es) without cCMV (cCMV-negative group), and multiple newborns/fetuses (eg, twins, triplets) with discordant CMV transmission (cCMV-mixed group).

The study was conducted in accordance with Good Clinical Practice guidelines and the Declaration of Helsinki and was approved by an independent ethics committee. Parent(s) gave additional informed consent for the recording of results of further testing of the newborn in case of live birth or the fetus in case of termination or stillbirth. The study was registered at www.clinicaltrials.gov (NCT01251744); a protocol summary is available at https://www.gsk-studyregister.com/en/trial-details/?id=113134.

### Assessments

Assessment of CMV infection diagnosis, safety, and symptomatic cCMV infections is described in the Supplementary Material.

Sampling points for the assessment of viral load and immune responses in pregnant women are shown in Supplementary Figure 1, with a minimum of 3 mandatory visits (study entry, pregnancy conclusion, and study conclusion). Viral load (expressed as the number of CMV DNA copies/mL) was measured by quantitative polymerase chain reaction (qPCR) in blood samples (plasma and buffy coat), saliva, urine, and vaginal secretions, at study entry and pregnancy conclusion. Anti-CMV immunoglobulin M (IgM) antibodies and anti-glycoprotein B (gB), anti-tegument, and anti-pentamer IgG antibodies were measured using enzyme-linked immunosorbent assay. CMV-specific neutralizing antibodies were measured using epithelial and fibroblast cell lines. Cell-mediated immunity (CMI) was assessed by intracellular cytokine staining and flow cytometry in terms of CMV-specific CD4^+^/CD8^+^ T-cell responses (Supplementary Material).

### Statistical Methods

#### Sample Size

Considering a target sample size of approximately 90 enrolled pregnant women with confirmed primary CMV infection and a maternal-fetal CMV transmission rate of 30%–40%, the number of infants born with cCMV infection was expected to range between 27 and 36.

#### Models for Correlates of Maternal-Fetal CMV Transmission

An initial univariate analysis was conducted to identify potential correlates of CMV transmission during pregnancy, but none of the analyzed parameters were individually predictive of risk of cCMV. Therefore, in a post hoc analysis, we developed alternative approaches looking into univariate or multivariate factors associated with cCMV status, including a participant clustering/stratification approach and an interpretable predictive model–based approach that includes or excludes cluster membership in trained decision trees for risk prediction. For simplicity, the women in the cCMV-mixed group were included in the cCMV-positive group in all analyses. As data collection started at the seroconversion timepoint (and not the beginning of the pregnancy), measurements of analyzed potential biomarkers of maternal-fetal CMV transmission at a particular timepoint (eg, study entry) were collected at different gestational ages for each participant. In all models, we thus included as variables either baseline characteristics independent of seroconversion time (except for gestational age at CMV diagnosis, which was used in the clustering approach), immunological biomarkers at the last sampling timepoint before delivery, or viral load data.

##### Analysis of Biomarkers for cCMV Prediction

Univariate/multivariate logistic regression models were used to identify biomarkers in pregnant women that predict CMV transmission to their fetus.

The measurements of humoral and CMI responses were scaled by the log transformation, and log-normalized values were used in fitting the linear models in the analysis. We also considered viral load data (as a measure of the progression of infection) and focused on measurements for urine and saliva samples as they tended to be above the detection level for most women included in the analyses. For each participant, we defined 4 statistic values per sample (mean and maximum value over all timepoints, and values at study entry and last collection timepoint before pregnancy conclusion) to summarize viral load.

##### Baseline Variables and Clustering Based on Dissimilarities

In the participant clustering/stratification model, we used as variables baseline demographic and behavioral characteristics of the mothers (having children <3 years of age, number of children, gestational age at diagnosis, occupational risk, living with a partner, smoking history [number of cigarettes per day], working with toddlers/children, fetus sex, multiple fetuses, number of previous live births, number of previous pregnancies, and age). We computed dissimilarity values between pairs of participants for the mixed data (including both continuous and categorical variables), using the Ahmad-Dey dissimilarity measure [17].

Embedding of dissimilarities was performed using the uniform manifold approximation projection (UMAP) method [18], which assumes the dissimilarities between participant data approximate distances between points (each representing a participant) on a 2D plane. Data points of very similar participants are placed close to each other, and those of dissimilar individuals are separated. However, UMAP embeddings do not provide any insight into the variable contributions.

We used a hierarchical clustering technique to identify clusters based on visual inspection and a chosen cut-off for clustering, which allows good cluster cohesion and a reasonable number and size for clusters.

##### Predictive Model for Maternal-Fetal CMV Transmission

Decision trees iteratively split an analysis into groups based on binary variables (via binarization of continuous variables) and evaluate the risk in that subgroup. To understand how the marker levels and baseline variables may be interacting and how to exploit the interactions for predictions, we trained decision trees on all data in the analysis set and visualized the decision flow. We trained decision tree models with 5 repeats of 10-fold cross-validation with up-sampling to keep the class probabilities of the data subset the same as original data. For each decision node, we represent the majority class and the fraction of cCMV-positive among all participants falling into that node.

Descriptive statistics of baseline characteristics of study participants and analyses of immune responses were performed using the Statistical Analysis System (SAS) v9.3 on SAS Drug Development 4.3.3 and STAT 12.1 for SAS. Statistical analyses to identify correlates of cCMV were executed using the R statistical computing environment (v4.1) and R packages (base R packages in addition to *dplyr*, *kmed*, *ggplot2*, and *umap* for visualization and *haven* for SAS file imports).

## RESULTS

### Study Participants

Of 82 women enrolled, 76 completed the study and 70 were included in the analyses: 22 (31.4%) in the cCMV-positive group, 45 (64.3%) in the cCMV-negative group, and 3 (4.3%) in the cCMV-mixed group (Supplementary Figure 2). Overall, baseline characteristics were similar between groups. CMV diagnosis was mostly based on appearance of CMV-specific IgG and IgM in blood in previously seronegative women (Table 1). Maternal signs of primary CMV infection were reported for 14 (63.6%), 28 (62.2%), and 3 (100%) participants in the cCMV-positive, cCMV-negative, and cCMV-mixed groups, respectively, with asthenia being the most common.

**Table 1.** Characteristics of Pregnant Women With Confirmed Primary Cytomegalovirus (CMV) Infection (Analysis Set) and Infants Enrolled in the Study (Enrolled Set), by Congenital CMV Status

Characteristic	cCMV Positive	cCMV Negative	cCMV Mixed
**Pregnant women**	n = 22	n = 45	n = 3
**Age at study entry, y, mean ± SD**	29.4 ± 6.2	29.6 ± 4.3	31.3 ± 1.5
***Geographic ancestry***			
White, Arabic/North African heritage	0 (0.0)	3 (6.7)	0 (0.0)
White, Caucasian/European heritage	22 (100)	42 (93.3)	3 (100)
**Gestational age at diagnosis, wk, mean ± SD**	17.9 ± 6.9	15.4 ± 7.5	18.3 ± 9.5
***Basis for CMV infection diagnosis***			
Appearance of IgG and IgM	14 (63.6)	30 (66.7)	3 (100)
Significant rise in IgG in the presence of IgM	5 (22.7)	9 (20.0)	0 (0.0)
Clinical symptoms	1 (4.5)	0 (0.0)	0 (0.0)
Missing data	2 (9.1)	6 (13.3)	0 (0.0)
**Cesarean delivery**	3 (18.8)	11 (22.4)	1 (33.3)
***Pregnancy outcome***			
Elective termination	3 (13.6)	0 (0.0)	0 (0.0)
Live birth	15 (68.2)	42 (93.3)	3 (100)
Spontaneous termination	1 (4.5)	0 (0.0)	0 (0.0)
Missing data	3 (13.6)	3 (6.7)	0 (0.0)
***Multiple birth***			
Yes	0 (0.0)	2 (4.4)	2 (66.7)
Missing data	3 (13.6)	3 (6.7)	0 (0.0)
***No. of pregnancies (including current)***			
1	3 (13.6)	14 (31.1)	1 (33.3)
2	12 (54.5)	17 (37.8)	1 (33.3)
3	2 (9.1)	10 (22.2)	1 (33.3)
4	4 (18.2)	3 (6.7)	0 (0.0)
≥5	1 (4.5)	1 (2.2)	0 (0.0)
***No. of previous live births***			
0	5 (22.7)	11 (24.4)	0 (0.0)
1	13 (59.1)	27 (60.0)	3 (100)
2	3 (13.6)	7 (15.6)	0 (0.0)
3	1 (4.5)	0 (0.0)	0 (0.0)
***No. of previous stillbirths***			
0	22 (100)	44 (97.8)	3 (100)
1	0 (0.0)	1 (2.2)	0 (0.0)
**Children aged <3 y**	16 (72.7)	30 (66.7)	2 (66.7)
**Regular close contact with toddlers**	8 (36.4)	21 (46.7)	1 (33.3)
**Working with infants/toddlers**	0 (0.0)	7 (15.6)	0 (0.0)
**Working in other risk occupation**	1 (4.5)	11 (24.4)	1 (33.3)
**Living with partner**	20 (90.9)	44 (97.8)	3 (100)
***Highest level of education***			
Higher education (≤3 y)	4 (18.2)	13 (28.9)	0 (0.0)
Higher education/university (>3 y)	8 (36.4)	21 (46.7)	2 (66.7)
Secondary school	8 (36.4)	9 (20.0)	1 (33.3)
Other	2 (9.1)	1 (2.2)	0 (0.0)
Missing data	0 (0.0)	1 (2.2)	0 (0.0)
***Daily cigarette smoking***			
0	21 (95.5)	40 (88.9)	3 (100)
1–5	1 (4.5)	4 (8.9)	0 (0.0)
11–15	0 (0.0)	1 (2.2)	0 (0.0)
**Infants**	n = 20	n = 58	
**Gestational age at birth, wk, mean ± SD**	38.3 ± 2.8	38.8 ± 2.4	…
**Female sex**	10 (50.0)	32 (55.2)	…
***Geographic ancestry***			
White, Arabic/North African heritage	0 (0.0)	2 (3.4)	…
White, Caucasian/European heritage	19 (95.0)	52 (89.7)	…
Other	1 (5.0)	4 (6.9)	…
**Breastfed**	15 (75.0)	53 (91.4)	…
***Vital signs, mean ± SD***			
Height, cm	48.7 ± 1.4	50.0 ± 2.6	…
Weight, kg	3.1 ± 0.6	3.3 ± 0.6	…
Cranial perimeter, cm	33.5 ± 2.1	34.2 ± 1.7	…
Apgar score	9.7 ± 0.6	9.5 ± 0.9	…

Data are presented as No. (%) unless otherwise indicated. Diagnosis of CMV infection was based on urine testing in all newborns except 1 in the cCMV-negative group. Additional testing of the amniotic fluid was performed in 11 (55.0%) and 36 (62.1%) newborns in the cCMV-positive and cCMV-negative group, respectively.

Abbreviations: CMV, cytomegalovirus; cCMV, congenital cytomegalovirus; cCMV-positive, mothers with 1/multiple newborn(s)/fetus(es) with congenital cytomegalovirus infection or infants with congenital cytomegalovirus infection; cCMV-negative, mothers with 1/multiple newborn(s)/fetus(es) without congenital cytomegalovirus infection or infants without congenital cytomegalovirus infection; cCMV-mixed, mothers with multiple newborns/fetuses with discordant congenital cytomegalovirus infection status; IgG, immunoglobulin G; IgM, immunoglobulin M; SD, standard deviation.

Of the 78 enrolled infants, 20 (25.6%) were cCMV positive and 58 (74.4%) cCMV negative. The infants’ baseline characteristics were similar between groups (Table 1). At the end of the 24 months of follow-up, 8 of 20 live-born babies with a confirmed cCMV infection were assessed as showing manifestations that were related/possibly related to CMV infection (Table 2). There was 1 neonatal death during the study, due to severe CMV infection and multiple complications of prematurity.

**Table 2.** Live-Born Children With Signs and Symptoms Considered to Be Related/Possibly Related to Congenital Cytomegalovirus Infection at the End of the 24-Month Follow-up

Live-Born Child	Pregnancy Conclusion (Month 0)	Month 1	Month 6	Month 12	Month 18	Study Conclusion (Month 24)
1^a^	Digestive tract and renal hyperechogenicity	Severe cCMV infection	Preterm birth; very low birth weight; periventricular cysts; possible cataract; hepatosplenomegaly; thrombocytopenia; petechia; respiratory distress	Died at 44 d	…	…
2^a^	Intracerebral lesions suggestive of cCMV infection	Subependymal cyst (frontal right); suspicion of cystic lymphangioma; thrombocytopenia	sign of cCMV	No sign of cCMV	Psycho-developmental delay; abnormal cerebral MRI (white-matter abnormalities)	Global psycho-developmental delay (expressive language delay and no walk)
3	Subependymal cysts	Subependymal cyst bilateral	No sign of cCMV	No sign of cCMV	No sign of cCMV	No sign of cCMV
4^a^	No sign of cCMV	Subependymal cyst and vasculitis; abnormal audiologic test; inguinal hernia with testicular torsion; dysmaturity	Severe bilateral hearing loss; psycho-developmental delay; axial hypotonia	Severe bilateral hearing loss; psycho-developmental delay; hypotonia; underweight; microcephaly	Chart review not done	Psycho-developmental delay; deafness; repeated infections; microcephaly; hypotonia
5^a^	No sign of cCMV	Subependymal/periventricular cyst unilateral	No sign of cCMV	Possible psycho-developmental delay	Psycho-developmental delay	Psycho-developmental delay
6	No sign of cCMV	Subependymal/periventricular cyst unilateral	No sign of cCMV	No sign of cCMV	No sign of cCMV	No sign of cCMV
7	No sign of cCMV	No sign of cCMV	No sign of cCMV	Possible hearing loss	Chart review not done	Language delay
8	No sign of cCMV	No sign of cCMV	No sign of cCMV	No sign of cCMV	Lost to follow-up	Language delay

The assessment of symptomatic cCMV infections, as well as of signs and symptoms considered to be related/possibly related to cCMV infection is described in the Supplementary Material.

Abbreviations: cCMV, congenital cytomegalovirus; MRI, magnetic resonance imaging.

^a^Manifestations in these children were considered serious at study conclusion.

### Biomarker Assessment

**Figure 1.**
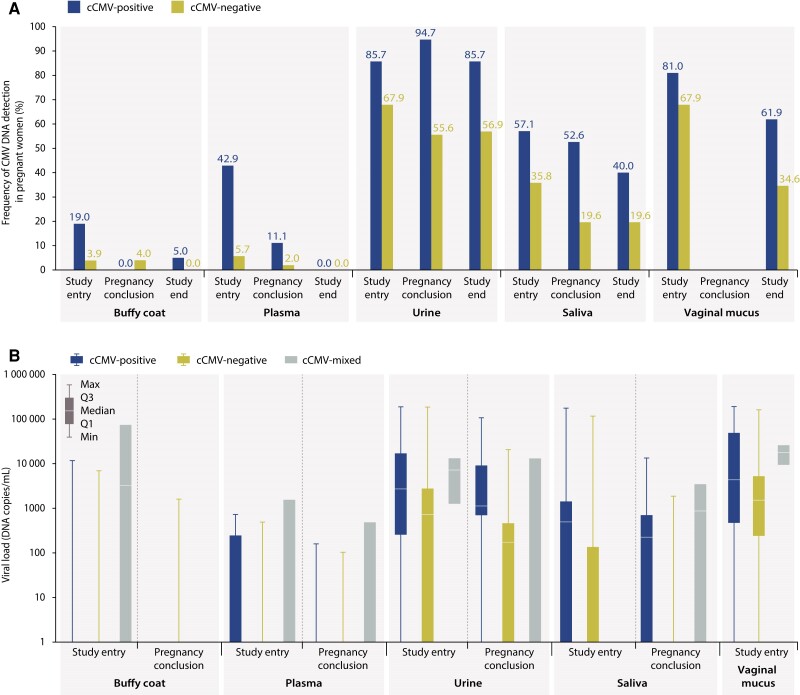
Frequency of cytomegalovirus (CMV) DNA presence (*A*) and viral load (*B*) in samples collected from pregnant women, by congenital CMV (cCMV) infection status and sample type (analysis set). Samples with DNA presence were samples with any DNA value >0, as established by quantitative polymerase chain reaction. In (*A*), the frequency is reported as the percentage of positive samples per visit calculated from all available samples per visit; mothers with multiple newborns with different cCMV status (cCMV-mixed group) were not included. In (*B*), boxplots represent the lower quartile (Q1), median, and upper quartile (Q3), and error bars represent the minimum and maximum values. Abbreviations: CMV, cytomegalovirus; cCMV, congenital cytomegalovirus; cCMV-positive, mothers with 1/multiple newborn(s)/fetus(es) with congenital cytomegalovirus infection; cCMV-negative, mothers with 1/multiple newborn(s)/fetus(es) without congenital cytomegalovirus infection; cCMV-mixed, mothers with multiple newborns/fetuses with different congenital cytomegalovirus infection status.

Very few pregnant women had detectable levels of CMV DNA in the buffy coat. The proportions of pregnant women with detectable CMV DNA (Figure 1*A*) and the median viral loads among CMV DNA–positive women (Figure 1*B*) were lower for plasma than for the other sample types.

Overall, throughout the pregnancy, seropositivity rates for anti-CMV neutralizing antibodies using epithelial or fibroblast cells were ≥90.5%, ≥97.4%, and ≥66.7% in the cCMV-positive, cCMV-negative, and cCMV-mixed groups, respectively (Table 3). Across groups, all women were seropositive for anti-pentamer, anti-gB, and anti-tegument IgG antibodies at least once during the study. Median avidity indexes for anti-gB and anti-tegument IgG antibodies at study entry and pregnancy conclusion were similar between groups (Supplementary Table 1).

**Table 3.** Seropositivity Rates, Geometric Mean Titer/Concentration (GMT/C), and Adjusted GMT/C for Anti-Cytomegalovirus (CMV) Neutralizing Antibodies Using Epithelial Cells and Fibroblast and for Anti-pentamer, Anti-gB, and Anti-tegument Immunoglobulin G Antibodies Throughout the Study, by Congenital CMV Infection Status (Analysis Set)

Group	Timing	no./No. (%) ≥ Cut-off^a^	GMT/C (95% CI)	Adjusted GMT/C (95% CI)
Anti-CMV neutralizing antibodies using epithelial cells (ARPE-19 cell line; cut-off: 15 ED_50_)				
cCMV-positive	Study entry	21/21 (100)	1082.7 (661.6–1772.0)	1183.8 (768.2–1824.2)
	Month 2	16/16 (100)	1276.3 (744.2–2188.9)	…
	Month 4	3/3 (100)	769.7 (23.6–25 108.6)	…
	Pregnancy conclusion	19/19 (100)	1250.3 (787.8–1984.4)	1143.1 (771.2–1694.2)
cCMV-negative	Study entry	43/44 (97.7)	720.6 (531.9–976.2)	690.5 (513.3–928.9)
	Month 2	38/39 (97.4)	892.5 (632.7–1259.0)	…
	Month 4	19/19 (100)	1254.2 (922.9–1704.5)	…
	Month 6	4/4 (100)	2137.1 (675.4–6762.2)	…
	Pregnancy conclusion	42/42 (100)	1035.1 (814.8–1315.0)	1078.0 (834.1–1393.2)
cCMV-mixed	Study entry	3/3 (100)	836.2 (131.2–5330.5)	…
	Month 2	1/1 (100)	785.0 (…)	…
	Month 4	1/1 (100)	703.0 (…)	…
	Pregnancy conclusion	3/3 (100)	1149.7 (705.8–1872.6)	…
Anti-CMV neutralizing antibodies using fibroblast cells (MRC-5 fibroblast cell line; cut-off: 10 ED_50_)				
cCMV-positive	Study entry	19/21 (90.5)	344.3 (163.8–723.6)	354.8 (197.5–637.5)
	Month 2	16/16 (100)	808.3 (507.0–1288.5)	…
	Month 4	3/3 (100)	872.1 (195.4–3891.6)	…
	Pregnancy conclusion	19/19 (100)	986.7 (703.7–1383.6)	983.9 (733.6–1319.5)
cCMV-negative	Study entry	43/44 (97.7)	263.5 (182.3–380.7)	259.7 (173.8–388.1)
	Month 2	38/39 (97.4)	364.3 (255.5–519.6)	…
	Month 4	19/19 (100)	386.6 (274.2–545.2)	…
	Month 6	4/4 (100)	550.3 (266.4–1136.8)	…
	Pregnancy conclusion	42/42 (100)	445.6 (376.7–527.1)	446.2 (368.5–540.2)
cCMV-mixed	Study entry	2/3 (66.7)	197.4 (.1–563 134.8)	…
	Month 2	1/1 (100)	338.0 (…)	…
	Month 4	1/1 (100)	266.0 (…)	…
	Pregnancy conclusion	3/3 (100)	927.5 (250.0–3440.6)	…
Anti-pentamer IgG antibodies (cut-off: 125.01 EU/mL)				
cCMV-positive	Study entry	19/20 (95.0)	13 000.6 (6163.6–27 421.7)	13 097.3 (7205.7–23 805.9)
	Month 2	16/16 (100)	20 083.5 (12 738.2–31 664.5)	…
	Month 4	3/3 (100)	11 322.8 (388.4–330 098.6)	…
	Pregnancy conclusion	19/19 (100)	20 254.1 (14 291.7–28 703.8)	18 907.2 (13 630.4–26 226.9)
cCMV-negative	Study entry	43/44 (97.7)	6405.3 (4396.1–9332.8)	6383.8 (4279.1–9523.8)
	Month 2	38/39 (97.4)	8225.1 (5713.1–11 841.7)	…
	Month 4	19/19 (100)	6568.6 (4821.7–8948.3)	…
	Month 6	4/4 (100)	8494.0 (4096.9–17 610.3)	…
	Pregnancy conclusion	39/39 (100)	9799.4 (7953.7–12 073.4)	10 133.5 (8119.6–12 646.9)
cCMV-mixed	Study entry	3/3 (100)	7729.0 (888.6–67 223.3)	…
	Month 2	1/1 (100)	6954.3 (…)	…
	Month 4	1/1 (100)	8403.5 (…)	…
	Pregnancy conclusion	3/3 (100)	19 457.8 (3747.9–101 018.0)	…
Anti-gB IgG antibodies (cut-off: 54 EU/mL)				
cCMV-positive	Study entry	21/21 (100)	5104.1 (3065.6–8497.9)	5259.5 (3312.7–8350.3)
	Pregnancy conclusion	19/19 (100)	8645.4 (6382.0–11 711.5)	8903.6 (6864.5–11 548.5)
cCMV-negative	Study entry	44/45 (97.8)	4424.6 (3197.5–6122.7)	4363.1 (3187.7–5972.1)
	Pregnancy conclusion	42/42 (100)	5963.8 (5140.1–6919.5)	5884.9 (4967.3–6972.1)
cCMV-mixed	Study entry	3/3 (100)	1962.7 (159.7–24 123.1)	…
	Pregnancy conclusion	3/3 (100)	6982.3 (928.3–52 520.2)	…
Anti-tegument IgG antibodies (cut-off: 0.668 EU/mL)				
cCMV-positive	Study entry	21/21 (100)	6.3 (4.1–9.5)	6.7 (4.5–9.8)
	Month 2	16/16 (100)	7.3 (4.6–11.6)	…
	Month 4	2/3 (66.7)	2.3 (.0–152.4)	…
	Pregnancy conclusion	19/19 (100)	6.6 (4.3–10.4)	6.5 (4.3–9.7)
cCMV-negative	Study entry	44/45 (97.8)	4.2 (3.2–5.5)	4.1 (3.1–5.3)
	Month 2	37/39 (94.9)	4.3 (3.1–6.0)	…
	Month 4	19/19 (100)	5.7 (3.8–8.6)	…
	Month 6	4/4 (100)	13.5 (3.9–46.2)	…
	Pregnancy conclusion	42/42 (100)	4.7 (3.6–6.1)	4.8 (3.7–6.2)
cCMV-mixed	Study entry	3/3 (100)	5.8 (1.0–35.1)	…
	Month 2	1/1 (100)	2.8 (…)	…
	Month 4	1/1 (100)	2.2 (…)	…
	Pregnancy conclusion	3/3 (100)	6.4 (.4–93.7)	…

Data were not available for all women at all time points due to differences in gestational age at study entry and/or due to attendance.

Antibody GMTs/Cs were calculated with 95% CIs by taking the anti-log of the mean of the log titer transformations/concentrations. For antibody titers below the assay cut-off, an arbitrary value of half the cut-off was used for GMT calculation. Adjusted GMTs/Cs were calculated using a general linear model with cCMV serostatus as a fixed factor and time of diagnosis (≤12 weeks or >12 weeks of pregnancy) and sample time since diagnosis (0–4 weeks, >4–8 weeks, or >8 weeks) as covariates.

Abbreviations: CI, confidence interval; CMV, cytomegalovirus; cCMV, congenital cytomegalovirus; cCMV-positive, mothers with 1/multiple newborn(s)/fetus(es) with congenital cytomegalovirus infection; cCMV-negative, mothers with 1/multiple newborn(s)/fetus(es) without congenital cytomegalovirus infection; cCMV-mixed, mothers with multiple newborns/fetuses with discordant congenital cytomegalovirus infection status; ED_50_, endpoint dilution 50%; EU, enzyme-linked immunosorbent assay units; gB, glycoprotein B; GMT/C, geometric mean titer/concentration; IgG, immunoglobulin G.

^a^Column shows number of samples above or equal to the assay cut-off/total number of samples.

**Figure 2.**
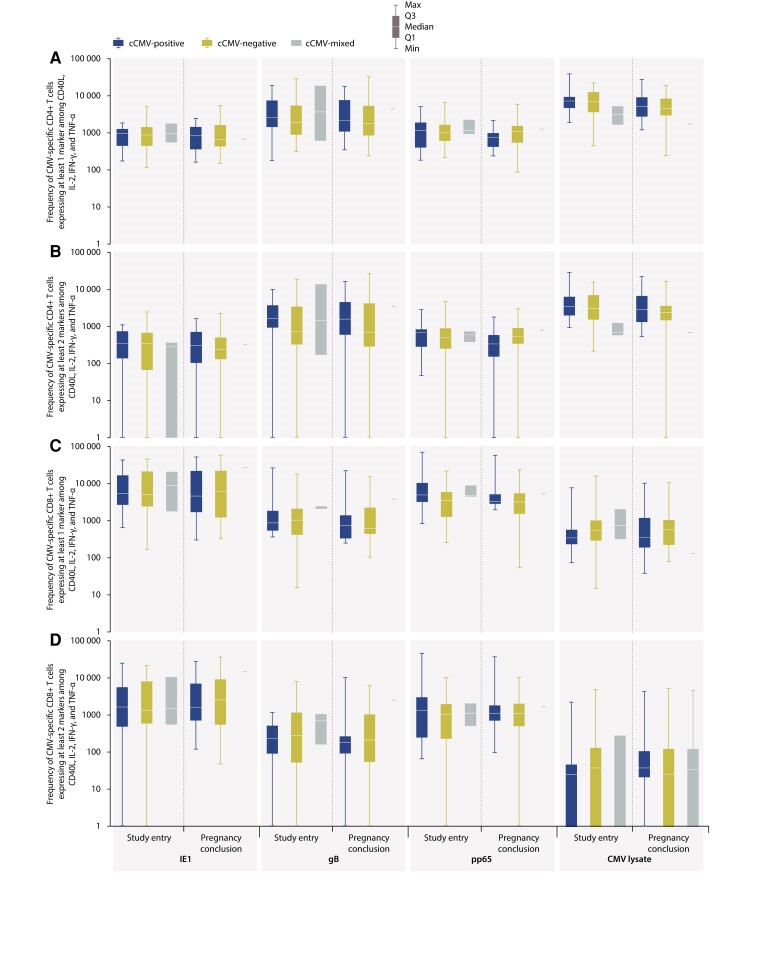
Frequency of cytomegalovirus (CMV)–specific CD4^+^ T cells (*A* and *B*) and CD8^+^ T cells (*C* and *D*) expressing at least 1 (*A* and *C*) or 2 (*B* and *D*) markers among CD40L, IL-2, IFN-γ, and TNF-α following stimulation with IE1, gB, pp65, and CMV lysate, at study entry and pregnancy conclusion, by congenital CMV (cCMV) infection status (analysis set). Box plots (lower quartile [Q1], median, and upper quartile [Q3]) with error bars (minimum and maximum) per group are shown. Data were calculated as the frequency of CMV-specific CD4^+^/CD8^+^ T cells expressing immune markers (number/10^6^ T cells) with 95% confidence interval. Each result was obtained by subtracting the background value (stimulation with medium only). Abbreviations: CMV, cytomegalovirus; cCMV, congenital cytomegalovirus; cCMV-positive, mothers with 1/multiple newborn(s)/fetus(es) with congenital cytomegalovirus infection; cCMV-negative, mothers with 1/multiple newborn(s)/fetus(es) without congenital cytomegalovirus infection; cCMV-mixed, mothers with multiple newborns/fetuses with different congenital cytomegalovirus infection status; CD40L, cluster of differentiation 40 ligand; gB, glycoprotein B; IE1, immediate-early 1 protein; IFN-γ, interferon gamma; IL-2, interleukin 2; pp65, phosphoprotein 65; TNF-α, tumor necrosis factor alpha.

The frequency of CMV-specific CD4^+^/CD8^+^ T cells expressing at least 1 or at least 2 markers among CD40 ligand, interleukin 2, interferon-γ, or tumor necrosis factor–α per 10^6^ CD4^+^/CD8^+^ T cells was similar between the cCMV-positive and cCMV-negative groups following stimulation with immediate-early 1 protein (IE1), gB, phosphoprotein 65 (pp65), or lysate of CMV-infected fibroblasts (CMV lysate), at study entry and pregnancy conclusion (Figure 2).

Although adjusted geometric mean concentrations/titers for anti-CMV neutralizing and anti-pentamer, anti-gB, and anti-tegument IgG antibodies at study entry and pregnancy conclusion tended to be higher in the cCMV-positive group, no consistent differences across timepoints were observed for immunological markers (Supplementary Table 2; Supplementary Figure 3).

### Models for Correlates of Maternal-Fetal CMV Transmission After Infection During Pregnancy

#### Clustering Based on Pairwise Dissimilarities at Baseline

**Figure 3.**
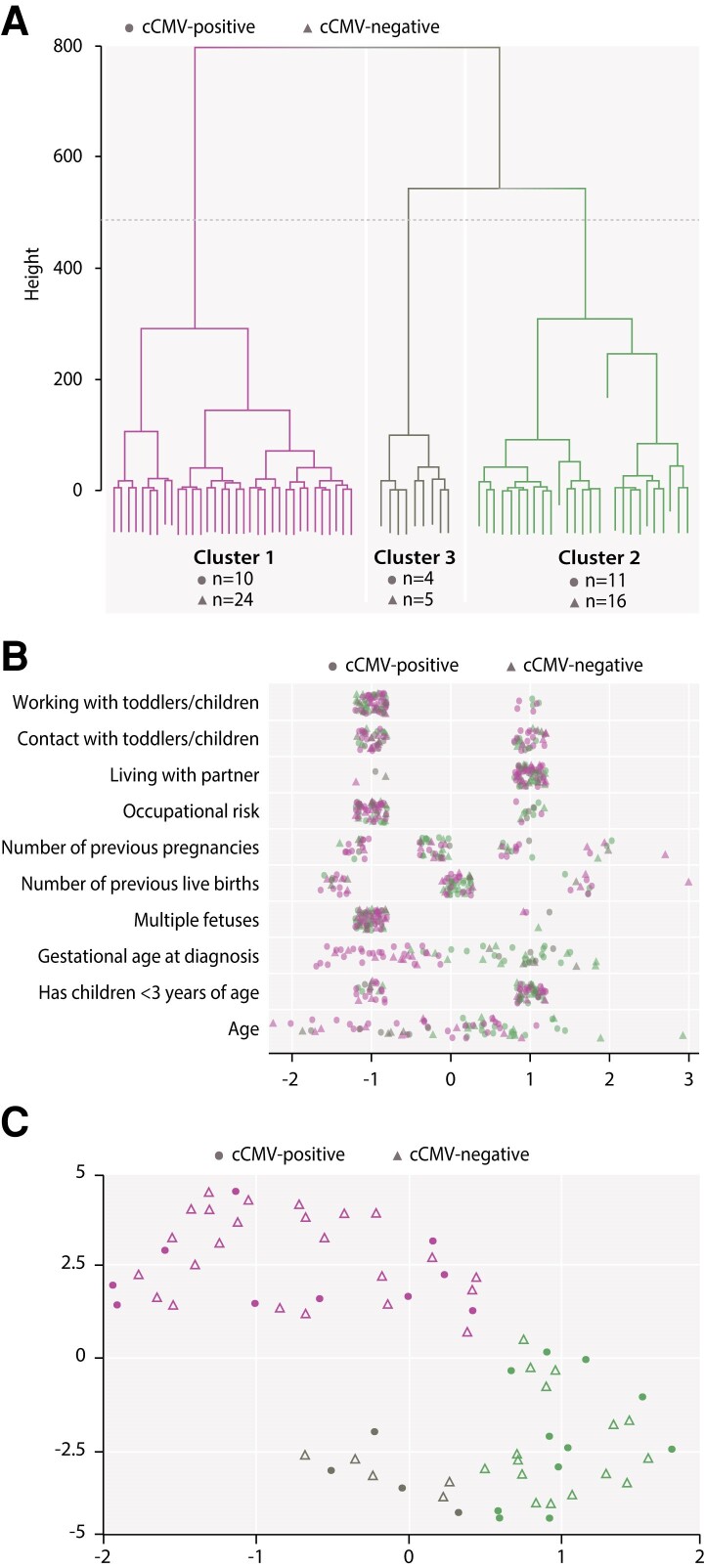
Hierarchical clustering of dissimilarities based on baseline variables (*A*), distribution of baseline variables based on clusters and congenital CMV (cCMV) status (*B*), and uniform manifold approximation projection (UMAP) embedding based on baseline variables (*C*). *A*, The number of clusters was chosen based on the trade-off between cluster size and cluster cohesion in a data-driven fashion. In the dendrogram plot, the y-axis shows the allowed maximum variability for a cluster (ie, incohesion). Clusters were split until only 1 example remained in each cluster (a single participant represented as the lines at bottom of the dendrogram). Based on visual inspection of the dendrogram, the horizontal dashed line was chosen as the cut-off point for clustering (providing good cluster cohesion, reasonable cluster size, and a small number of clusters). *B*, Noise was added to point coordinates to avoid overplotting. Values for binary variables were recoded as −1 and 1. Numeric variables were standardized to have 0 mean and standard deviation of 1. Number of pregnancy values were originally 1, 2, 3, and 5, and number of live birth values were 0, 1, 2, and 3. Age values (19 [min], 29.73 [mean], and 44 [max]) have been scaled to −2.21, 0.0, and 2.94, respectively; 1 unit in the plot x-axis corresponds to 4.85 years. Gestational age at time of infection values (4 [min], 16.3 [mean], and 30 [max]) have been rescaled to −1.67, 0.0, and 1.86; 1 unit in the plot x-axis corresponds to 7.4 weeks. *C*, The UMAP embedding coordinates and their units do not correspond to meaningful quantities as the particular coordinates are determined only up to an arbitrary rotation. The global shape and point distribution and clustering provide insights for UMAP embeddings. Abbreviations: CMV, cytomegalovirus; cCMV, congenital cytomegalovirus; cCMV-positive, mothers with 1/multiple newborn(s)/fetus(es) with congenital cytomegalovirus infection (including mothers with multiple newborns/fetuses with different congenital cytomegalovirus infection status); cCMV-negative, mothers with 1/multiple newborn(s)/fetus(es) without congenital cytomegalovirus infection.

We identified 3 clusters (Figure 3*A*), including 34 (cluster 1; 10 cCMV-positive and 24 cCMV-negative), 27 (cluster 2; 11 cCMV-positive and 16 cCMV-negative), and 9 (cluster 3; 4 cCMV-positive and 5 cCMV-negative) women. We plotted the distribution of baseline characteristics based on clusters and cCMV status and found that gestational age at diagnosis (with clusters 2 and 3 showing a tendency for infection later in pregnancy) and, to a lesser extent, age (women in cluster 2 tended to be older than those in clusters 1 and 3) had a larger contribution to the clustering (Figure 3*B*). Based on the visualization of the 2D embedding, we identified a clear separation between cluster 1 and the other clusters, while cluster 3 can be considered an outgrowth of cluster 2 (Figure 3*C*).

#### Potential Biomarkers for cCMV Prediction

In univariate analyses, we observed a trend for higher viral load measurements in the cCMV-positive group than the cCMV-negative one, most prominent in urine samples throughout the study and at study entry for saliva samples (Supplementary Figure 4). This was largely confirmed by Wilcoxon rank-sum 2-sided statistics (Supplementary Table 3).

We visually inspected violin plot representations of viral load values for each cluster. We found that participants in cluster 3 tended to have above-average viral load statistics, most apparent in the plots for maximum viral load in urine samples and viral load at study entry in saliva samples (Supplementary Figure 5).

In univariate analyses conducted for the last sample collection timepoint before delivery, no statistical correlation between any immunological biomarker and cCMV status was identified, except for CMV-specific CD8^+^ T cells expressing at least 2 immune markers following stimulation with CMV lysate. By contrast, we observed a trend for higher likelihood of cCMV with higher anti-pentamer IgG antibody concentration and fibroblast neutralizing antibody titer levels (Supplementary Figure 6).

Using multivariate logistic regression, we identified 1 humoral and 2 CMI-based immunological biomarkers with predictive power for maternal-fetal CMV transmission: the anti-pentamer IgG antibody concentration and the frequency of CMV-specific CD4^+^ and CD8^+^ T cells expressing at least 2 immune markers following stimulation with CMV lysate and IE1, respectively. Two other antibody levels may be relevant for the CMV transmission risk, showing borderline association with cCMV status: the anti-tegument and anti-gB IgG antibody levels (Supplementary Table 4).

#### Predictive Model for Maternal-Fetal CMV Transmission

**Figure 4.**
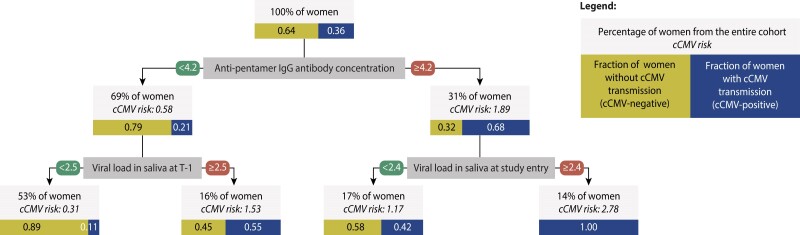
Decision tree trained on all cohort data that include cluster membership variables, viral load, and humoral and cell-mediated immunity–based biomarker variables. In the decision tree, the cohort underwent successive binary divisions based on the considered variables until, to the extent feasible, the subnodes at the bottom contained predominantly either women transmitting congenital cytomegalovirus (cCMV) (cCMV-positive) or not transmitting cCMV (cCMV-negative). The tree was trained on all variables, but only 3 were retained. For each node and subnode, the percentage of women from the entire cohort at node, the fold-change in risk as compared with the entire cohort, and the fraction of cCMV-positive or cCMV-negative women are shown. Abbreviations: CMV, cytomegalovirus; cCMV, congenital cytomegalovirus; cCMV-positive, mothers with 1/multiple newborn(s)/fetus(es) with congenital cytomegalovirus infection (including mothers with multiple newborns/fetuses with different congenital cytomegalovirus infection status); cCMV-negative, mothers with 1/multiple newborn(s)/fetus(es) without congenital cytomegalovirus infection; CMI, cell-mediated immunity; IgG, immunoglobulin G; T-1, last sampling time before delivery.

Finally, we used the combined participant clustering, viral load, and immunological biomarkers to train predictive models of cCMV. Anti-pentamer IgG antibody concentration and viral load measurements in saliva were the variables retained in the final decision tree trained on all variables. The cohort at the root of the decision tree is split according to a threshold of 4.2 for a normalized anti-pentamer IgG antibody concentration (ie, 15 849 enzyme-linked immunosorbent assay units/mL). Higher anti-pentamer IgG antibody levels are indicative of a 3-fold increase in the risk of cCMV compared with levels under the threshold. The second set of splits is based on very low levels of 2 viral load statistics in saliva samples; the risk of cCMV increases with increased viral load levels (Figure 4).

## DISCUSSION

In this descriptive study, we investigated correlations between virologic or immunologic parameters measured in pregnant women with confirmed primary CMV infection and transmission of CMV infection to the fetus. Since primary CMV infection resulted in congenital infection in 25 of 70 women included in the analyses, the infection rate in our study is around 36%, similar to findings from previous studies [19–21].

cCMV risk profiles were identified by clustering the study participants according to baseline demographic and behavioral characteristics. The pregnant women clustered in 3 distinct subgroups and cluster membership was strongly correlated with the gestational age at diagnosis, with higher gestational age tending to increase cCMV risk, in line with previous findings [22]. While the relatively low sample size precluded further analysis in our study, clustering provides an additional risk stratification that can improve predictive models [23].

Viral load was shown to be higher in women from the cCMV-positive group, indicating that poor control of viral replication at the systemic level may lead to CMV transmission, in line with findings from a previous study [19]. As expected, during the entire study period, almost all women in the study were seropositive for anti-pentamer IgG, anti-CMV tegument, and anti-gB antibodies, indicative of the broad antibody response mounted against primary CMV infection [24]. Women in the cCMV-positive group had higher anti-pentamer IgG antibody concentration at pregnancy conclusion than those in the cCMV-negative group, when adjusting for gestational age at diagnosis and sample collection time after diagnosis. Anti-pentamer IgG antibody response in pregnant women was previously found to be associated with reduced vertical CMV transmission [13, 14], but other studies suggest that neutralizing antibodies targeting the pentameric complex do not correlate with cCMV risk [25]; however, these studies did not consider interaction effects between potential immunomarkers. We found a correlation between cCMV risk and the frequency of CD4^+^ T cells expressing at least 2 immune markers following stimulation with IE1, but also for CD8^+^ expressing at least 2 immune markers following stimulation with CMV lysate. The latter finding is somewhat surprising considering that CMV lysate is primarily a stimulator of CD4^+^ and not CD8^+^ T cells [26] as it does not contain peptides of defined length to bind human leukocyte antigen class I molecules [27]. Of note, in a recent study conducted in 60 pregnant women, no association was found between CD4^+^ or CD8^+^ T-cell response at the time of maternal primary CMV infection and the risk of cCMV [28]. However, previous studies in nonhuman primates showed that both CD4^+^ and CD8^+^ T cells may protect against cCMV [29]. Moreover, in another study, high CMI responses, of which the majority were CMV pp65-specific CD3^+^CD8^+^ interferon-γ^+^ T cells, were shown to be a predictor of cCMV transmission, especially when used in combination with low CMV IgG avidity [30].

Univariate analyses in our study showed no clear association between the evaluated biomarkers and transmission of CMV from the mother to the fetus. In fact, so far, no single clinical or biological factor has been shown to be consistently associated with cCMV [31]. In view of the complexity of immune responses elicited by primary maternal infection and the differences in baseline characteristics of pregnant women, it is reasonable to assume that the correlate(s) of vertical CMV transmission may be a composite one. However, even after adjusting biomarker values for gestational age at sample collection, interaction effects between baseline variables and immunologic biomarkers would not be captured by univariate, or possibly even multivariate, analyses. We therefore constructed a predictive model using trained decision trees, which could account for the interplay of several potential biomarkers predictive of cCMV infection.

Our predictive model shows that women with low anti-pentamer IgG antibody levels throughout pregnancy and low viral load in saliva just before delivery (indicating good viral clearance) are the least likely to transmit CMV to their infants. An increased risk is predicted for women with relatively low anti-pentamer IgG antibody levels but high viral load in saliva just before delivery. This may be the case when the mother did not mount a proper immune response, thus increasing the risk of CMV transmission. Our model also indicates that sustained anti-pentamer IgG antibody levels throughout the study (indicating immunity) were associated with an increased risk of cCMV. The risk is the highest for women with high viral shedding through saliva early in pregnancy, as shown by the predictive value of saliva viral load at enrollment in our study. This may be indicative of a strong, systemic infection that leads to the mounting of a strong immune response throughout the pregnancy.

Our study and predictive model are not without limitations. The study was conducted in generally healthy pregnant women of predominant White–Caucasian/European heritage from a high-income country. Therefore, our findings may not be generalizable to other populations. The study enrolled a relatively low number of women, and thus independent validation of our predictive model is needed. This also applies to the thresholds for the predictive variables identified, which are inherent to our study and cannot be generalized to other cohorts. Studies with a larger sample size and wider geographic diversity are needed to establish accurate thresholds for the predictive biomarkers. Moreover, the timing of seroconversion and delivery differed between the pregnant women included in the study, and the collection of biomarker data occurred at different gestational ages. This heterogeneity could have led to overlooking possible associations between the study variables and the outcome because the data have additional variance due to the immune response kinetics. In addition, assessment of biomarkers was not exhaustive; for instance, antibody avidity [32] or antibody-dependent cellular phagocytosis [15], which have been previously suggested to correlate with CMV transmission, were not considered in our analysis.

Using a predictive-model approach, we identified anti-pentamer IgG antibody concentration during pregnancy and viral load in saliva samples as biomarkers jointly associated with the risk of cCMV infection. Validation of these biomarkers in future studies will guide the management of primary maternal infection and the development of vaccines against cCMV.

## Supplementary Data

Supplementary materials are available at *The Journal of Infectious Diseases* online (http://jid.oxfordjournals.org/). Supplementary materials consist of data provided by the author that are published to benefit the reader. The posted materials are not copyedited. The contents of all supplementary data are the sole responsibility of the authors. Questions or messages regarding errors should be addressed to the author.

## Supplementary Material

jiae281_Supplementary_Data

## Notes

***Acknowledgments.*** The authors thank the study participants and site staff involved in the study. The authors are grateful to Dominique Thomas (Hôpitaux Iris Sud–site Ixelles, Brussels, Belgium) for her contribution as a study investigator and Olivier Gruselle, Thierry Pascal (GSK), Dirk van Alewijk, Leontine van der Wel, and Ilona van Haaften (Viroclinics-DDL) for their help with qPCR characterization and clinical testing. The authors also thank Akkodis Belgium c/o GSK for medical writing (Petronela M. Petrar, Lea El Asmar), design support, and manuscript coordination.

***Author contributions.*** Conceptualization/design of the study: A. M., I. D., and C. D. Data collection/generation: A. M., X. C., N. D. S., J.-M. D., R. D., I. D., C. D., M. J., P. L., and R. P. Performed the study: A. M., J.-M. D., I. D., P. L., and R. P. Contributed with materials/analysis tools: A. M., S. A., J.-M. D., and R. P. Data analysis/interpretation: A. M., S. A., H. A., V. B., N. D. S., R. D., M. J., A. A. P., C. S., R. A. v. d. B., and R. P. Writing—original draft: A. M., S. A., H. A., V. B., and R. A. v. d. B. Review and editing: All authors.

***Data availability.*** Anonymized individual participant data and study documents can be provided upon request from www.clinicalstudydatarequest.com.

***Financial support*.** This work was funded by GlaxoSmithKline Biologicals SA, including all costs associated with the development and publication of this manuscript.
