# Supplementary material for: Establishing Correlates of Maternal-Fetal Cytomegalovirus Transmission—One Step Closer Through Predictive Modeling
Source: J Infect Dis. 2024 Jun 12;230(6):e1274–86. doi: 10.1093/infdis/jiae281 (PMC11646613; doi:10.1093/infdis/jiae281)
Supplement: jiae281_Supplementary_Data [file jiae281_supplementary_data.docx]

Supplementary Materials

**List of Supplementary Materials**

Exclusion criteria for enrollment in the study

Diagnosis of (congenital) CMV infection

Assessment of safety and symptomatic cCMV infections

Assessment of viral load and immune responses

Supplementary Figures 1–6

Supplementary Tables 1–4

Exclusion criteria for enrollment in the study

Pregnant adult women were excluded from the study in case of chronic administration (defined as more than 14 days) of immunosuppressive or other immune-modifying drugs within six months prior to study entry, receipt of previous investigational cytomegalovirus (CMV) vaccine, concurrent participation in another clinical trial in which the participant had been exposed to an investigational or a non-investigational pharmaceutical product, any confirmed or suspected immunosuppressive or immunodeficient condition, major congenital defects, serious chronic illness, organ transplantation, administration of immunoglobulins and/or blood products within the three months preceding study enrollment or during the pregnancy (excluding at the time of delivery) with the exception of Rho(D) immune globulin products, documented human immunodeficiency virus infection, and a gestational age of more than 34 weeks as determined by fetal ultrasound.

Diagnosis of (congenital) CMV infection

Primary CMV infection was confirmed in pregnant women (1) by appearance of CMV-specific immunoglobulin (Ig) G and IgM antibodies in women who were CMV seronegative before pregnancy or at the onset of pregnancy, (2) by a significant rise in CMV-specific IgG antibody concentrations in the presence of CMV-specific IgM antibodies, or (3) by clinical symptoms/signs and/or biological alterations compatible with CMV infection in the presence of CMV-specific IgM antibodies without evolution of CMV-specific IgG antibodies.

In case of a live birth, diagnosis of congenital CMV (cCMV) was based on assessment of the urine. For abortion or stillbirth, the diagnosis was based on tissue analysis of the fetus. If these assessments were not performed, analysis was based on testing of amniotic fluid.

Assessment of safety and symptomatic cCMV infections

Serious adverse events related to study participation (e.g., protocol-mandated procedures, invasive tests, a change from existing therapy) or any fatalities were recorded for the entire study duration.

The occurrence of CMV-related signs and symptoms in CMV-infected children or fetuses as ascertained by the investigator upon medical record review and structured interview of the pediatrician or obstetrician was recorded. Each sign/symptom was classified based on causal relation to CMV infections as detailed below by three consensus pediatricians:

- Related to cCMV infection: any sign/symptom (i) part of a pre-defined list of signs/symptoms classically described as being associated with cCMV infection (see below), or (ii) judged as likely to be associated with cCMV infection based on previously published data and for which there was no valid alternative cause.
- Possibly related to cCMV infection: any sign/symptom for which (i) insufficient elements were available to make a judgement, or (ii) there was no consensus.
- Not related to cCMV infection: any sign/symptom for which a causal relation to cCMV infection was judged as absent.

The pre-defined list of signs/symptoms classically described as being associated with cCMV infection was:

1. Fetal signs associated to CMV infection:^1^
   1. Abnormality except those for the central nervous system (CNS): intrauterine growth restriction (IUGR); hepatic calcifications; ascites and/or pleural effusion; fetal hydrops; oligo or polyhydramnios; digestive tract hyperechogenicity; placental enlargement
   2. CNS abnormality (ultrasound/magnetic resonance imaging): microcephaly; subepandymal cysts; abnormal white matter and periventricular; cerebral calcifications; cerebral ventriculomegaly; lenticular striated vasculopathy (LSV); intraventricular abnormality; thalamic hyper-echogenicity; cerebellar abnormalities
2. Signs and symptoms at birth (a child was considered as symptomatic if there were clinical signs and/or biological signs and/or abnormal imagery with signs associated to cCMV):

a. Abnormality except those for the CNS: prematurity defined as <38 weeks of gestation;^2^ IUGR;^2-5^ hepatomegaly/splenomegaly;^2-5^ petechia;^2-5^ jaundice;^2-5^ thrombocytopenia (<100000/µL); ^2-4^ hepatitis with increase of hepatic transaminases (aspartate aminotransferase or alanine aminotransferase >80 U/L) and hyperbilirubinemia (bilirubin concentration >3 mg/dL); ^2-4^ pneumonia;^2^ anemia (hemolytic);^2^ inguinal hernia in boys;^2^ purpura;^2^ colitis, esophagitis^2^

b. Neurologic findings: microcephaly (head circumference less than the 3^rd^ percentile or more than 2 standard deviations of the norm);^2,4^ hypotonia, lethargy;^2,4^ hearing loss;^2,4^ abnormalities at cranial ultrasound or cerebral computed tomography scan/magnetic resonance imaging (defined by intracranial calcifications, cerebral ventriculomegaly, cysts, LSV, abnormal white matter, migrational abnormalities [lissencephaly, polymicrogyria]; cerebellar hypoplasia, corpus callosum dysgenesis); increased cerebrospinal fluid protein; polymerase chain reaction (PCR) positive for CMV in the cerebrospinal fluid); seizures;^2^ poor suck; chorioretinitis and /or optic atrophy;^2^ hydrocephaly^2^

3. Signs and symptoms beyond neonatal period:^2^ ocular defects (strabismus, optic atrophy, retinal necrosis, calcification, blindness, anterior chamber and optic disk malformation); deafness; dental defects; mental retardation; microcephaly, paralysis, seizures; psychomotor development delay; expressive language delay without hearing loss and mental impairment; hepatic calcifications.

References:

1. Benoist G, Salomon LJ, Mohlo M, Suarez B, Jacquemard F, Ville Y. Cytomegalovirus-related fetal brain lesions: comparison between targeted ultrasound examination and magnetic resonance imaging. Ultrasound Obstet Gynecol **2008**; 32:900-5.
2. Britt WJ. Cytomegalovirus. In: Remington JS, Klein JO, eds. Infectious diseases of the fetus and newborn infant. 7th ed. Philadelphia: Elsevier Saunders, **2011**.
3. Boppana SB, Pass RF, Britt WJ, Stagno S, Alford CA. Symptomatic congenital cytomegalovirus infection: neonatal morbidity and mortality. Pediatr Infect Dis J **1992**; 11:93-9.
4. Noyola DE, Demmler GJ, Nelson CT, et al. Early predictors of neurodevelopmental outcome in symptomatic congenital cytomegalovirus infection. J Pediatr **2001**; 138:325-31.
5. Gandhi RS, Fernandez-Alvarez JR, Rabe H. Management of congenital cytomegalovirus infection: an evidence-based approach. Acta Paediatr **2010**; 99:509-15.

Assessments of viral load and immune responses

*Viral load*

Viral load (expressed as the number of CMV DNA copies/mL) was measured in pregnant women by quantitative PCR (qPCR) in blood samples (plasma and buffy coat [peripheral blood leukocytes]; every two months), saliva, urine, and vaginal secretions (every month) from study entry to, and including, pregnancy conclusion, and at one-month post-pregnancy conclusion. The CMV DNA detection test used was designed to detect the phosphoprotein 65 (pp65) gene and amplification was based on the TaqMan probe technology, as previously described in detail in Paris R, Apter D, Boppana S, et al. Incidence of Cytomegalovirus Primary and Secondary Infection in Adolescent Girls: Results From a Prospective Study. J Infect Dis **2023**; 228:1491-95. We also estimated the number and proportion of samples positive (qPCR result >0) for CMV DNA per visit.

*Immunological biomarkers*

The humoral and cellular immune responses to CMV were assessed in pregnant women from blood samples collected every two months from study entry to, and including, pregnancy conclusion.

1. Anti-CMV tegument proteins IgG enzyme-linked immunosorbent assay (ELISA) (Biotest anti-CMV recombinant IgG ELISA)

This test is an indirect solid-phase ELISA for the detection of specific IgG antibodies using two highly purified, autologous fusion proteins, CG1 and CG2, each combining two immunodominant fragments from human CMV tegument (pp150; CG1:UL32, amino acids [aa] 495-691 and 862-1048; CG2:UL32, aa 695-854) and the delayed-early DNA binding protein (pp52; UL44, aa 297-433). If the sample contains specific antibodies, these bind to the recombinant antigens in the microtiter wells. Non-specific antibodies were removed during the first washing step. The resulting antibody-antigen complex was detected by peroxidase-labelled monoclonal anti-human IgG antibodies. The presence of bound antibodies was demonstrated by adding the chromogen-substrate solution (tetramethylbenzidine [TMB] and H_2_O_2_). The cut-off was determined by adding 0.3 to the mean optical density (OD) value of the negative control. Samples with index values ranging between 0.9 and 1.0 were considered as borderline.

2. Anti-CMV microneutralization assay

CMV neutralization assays on fibroblast (MRC-5, ATCC # CCL-171) and epithelial cells (ARPE-19, ATCC # CRL-2302) were used to quantify CMV neutralizing antibodies in human serum by assessing their ability to inhibit the CMV infection on cells. The two assays were used because the infection of fibroblasts and epithelial cells involve different viral glycoproteins, with epithelial cell infection also involving the CMV pentamer. AD169 and TB40/E strains were used to infect fibroblast and epithelial cells, respectively. Sera, after being heat-inactivated, were 2-fold serial diluted in a 96-well plate. Once diluted, a standardized amount of replication-capable virus (100 infectious particle unit [IPU]) was applied to the plate and incubated with serum to allow binding of the antibodies to the virus. After incubation (1 h), the serum-virus complex was transferred to a 96-well plate containing cells and plates were centrifuged (1 h, 2000 rpm, room temperature). Once completed, the medium with 10% of decomplemented fetal bovine serum was added in all the wells and the plate was incubated at 37°C for 24 h. After the incubation, cells were fixed and permeabilized with 4% paraformaldehyde, and 0.2% Triton-X100 for MRC-5 or with cold 80% acetone for ARPE-19. The staining was done with anti- immediate-early 1 protein (IE1) biotinylated monoclonal antibody and IPU counts were read with an automated reader. The serum neutralizing antibody titer was reported as the inverse of the serum dilution which yields a 50% reduction in the number of IPU compared to the virus control without serum (endpoint dilution 50).

3. Anti-glycoprotein B (gB) IgG ELISA

Anti-CMV gB antibody concentrations were measured by ELISA using recombinant gB as coating antigen on 96-well microplates. After washing, the wells are blocked with bovine serum albumin. Two dilutions of sera, in duplicate, as well as controls were incubated onto the coated wells to allow the specific antibodies, present in the sample, to react with gB. Non-specific reactants were removed by washing, and peroxidase-conjugated anti-human IgG polyclonal antibodies were added to bind with anti-gB IgG antibodies. Excess conjugate was removed by washing. Enzyme substrate solution (TMB) was added, and the blue color was allowed to develop. The reaction was stopped by addition of sulphuric acid, resulting in a color change to yellow. The intensity of the color was quantified and expressed in ELISA units (EU)/mL. The OD is proportional to the concentration of anti-gB antibodies present in the sample. Titers of each serum dilution were calculated by reference to a standard serum using the 4-parameters equation (mean OD), and the final titer of a serum was the mean of the titers falling in the proportional part of the reference curve.

4. Avidity of anti-gB antibodies

This assay was performed according to de Souza et al. (de Souza S, Bonon SH, Costa SC, Rossi CL. Evaluation of an in-house specific IgG avidity ELISA for distinguishing recent primary from long-term human cytomegalovirus (HCMV) infection. Rev Inst Med Trop Sao Paulo. **2003**; 45:323-6) using an elution step with urea to remove low-avidity antibodies from gB antigen. Briefly, each diluted serum was first added to wells of plates coated with gB antigen. After incubation, the unbound antibodies were removed by washing, and 8M urea in phosphate buffered saline was then added in the coated wells to dissociate low-avidity antibodies. The microplate was then washed, and horseradish peroxidase (HRP)-conjugated anti-human IgG antibodies were added. After incubation, unbound antibodies were removed by washing and TMB was added to reveal the enzyme activity. The color reaction was stopped by addition of sulphuric acid and the resulting yellow color was measured spectrophotometrically. The avidity index was calculated as the mean absorbance of reactions in which the immune complexes are exposed to urea divided by the mean absorbance of reactions in which the immune complexes are not exposed to urea, expressed as a percentage.

5. Cell-mediated immune responses

Cell-mediated immune responses were assessed by intracellular cytokine staining and flow cytometry in terms of CMV-specific CD4+/CD8+ T cell responses. The test assessed the frequency of IL-2, IFNγ, TNFα, and CD40L single and double positive CD4+ and CD8+ T cells. Peripheral blood mononuclear cells (PBMC) were cultured in the presence or absence of antigen preparations or peptides (CMV lysate, gB, IE1, or pp65) for 20 hours. To inhibit cytokine secretion, an intracellular blocking agent (GolgiPlug [BD Biosciences] or Brefeldin A [Roche Diagnostics]) was added for the last 18 hours of stimulation. Cell membranes were labelled with fluorescent anti-CD4 and anti-CD8 monoclonal antibodies. Following permeabilization, intracytoplasmic staining was performed using fluorescent anti-IL-2, anti-IFNγ, anti-TNFα, and anti-CD40L monoclonal antibodies. Labelled cells were quantitated by flow cytometry.

Proliferation of CMV-specific CD4+ T cells was measured by bromodeoxyuridine (BrdU) staining and flow cytometry. PBMCs were cultured in the presence and absence (background) of antigen preparations or peptides for six days. Cells were pulsed overnight with BrdU, the plasma membrane was labelled with fluorescent anti-CD4 monoclonal antibodies, and upon permeabilization, fluorescent anti-BrdU monoclonal antibodies were applied. Labelled cells were quantitated by flow cytometry.

All laboratory assays were performed in a validated laboratory designated by GSK using standardized procedures.

## Supplementary Figure 1. Study design


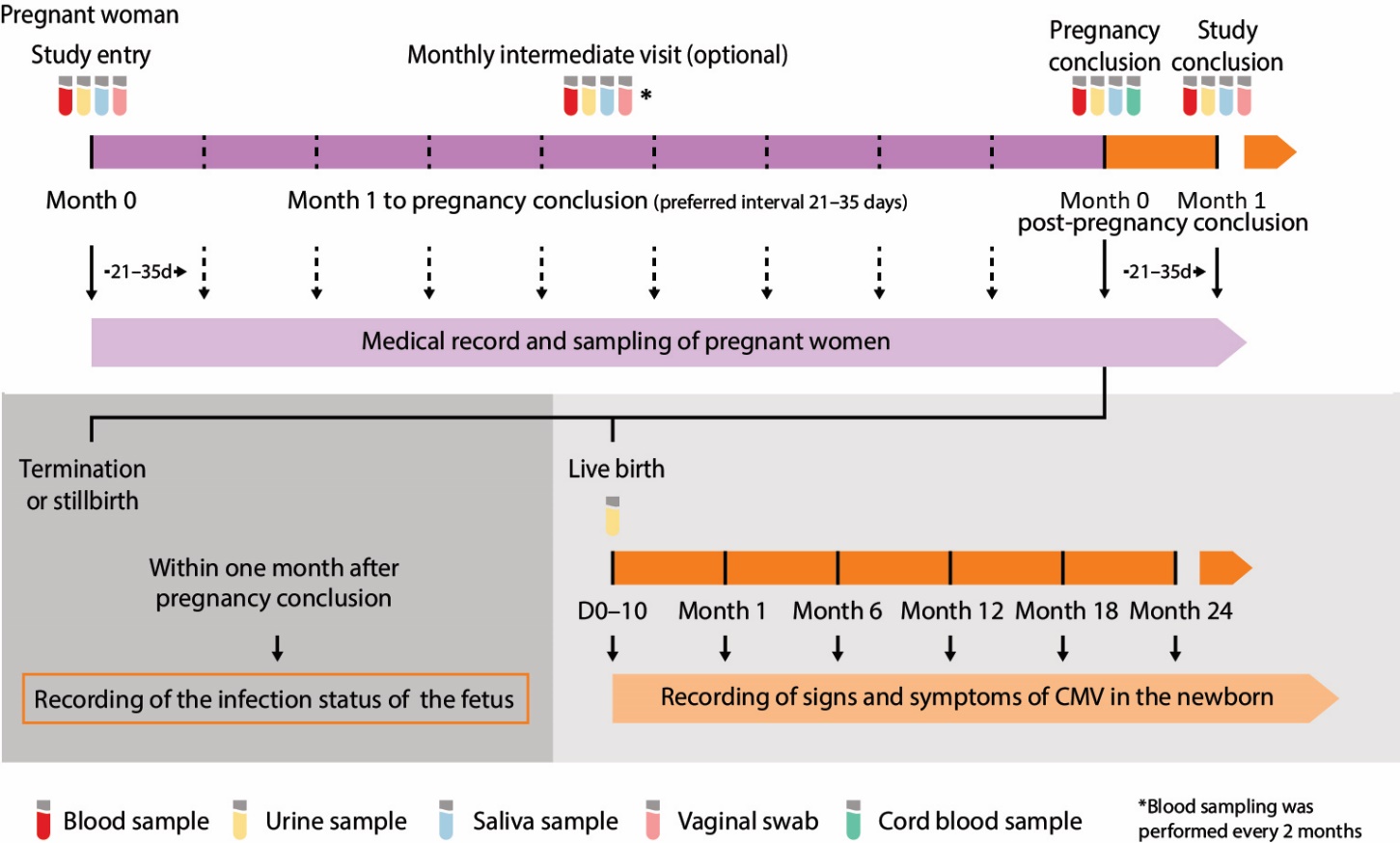


CMV, cytomegalovirus; D, day.

Note: Study entry corresponded to CMV diagnosis for each participant. The number of visits varied depending on the week of pregnancy of the participant when she entered the study. There was a minimum of three visits for every participant (study entry, pregnancy conclusion and study conclusion).

The recording of signs of symptoms of CMV was only applicable to newborns with confirmed congenital CMV infection (cCMV-positive group).

## Supplementary Figure 2. Participant flowchart


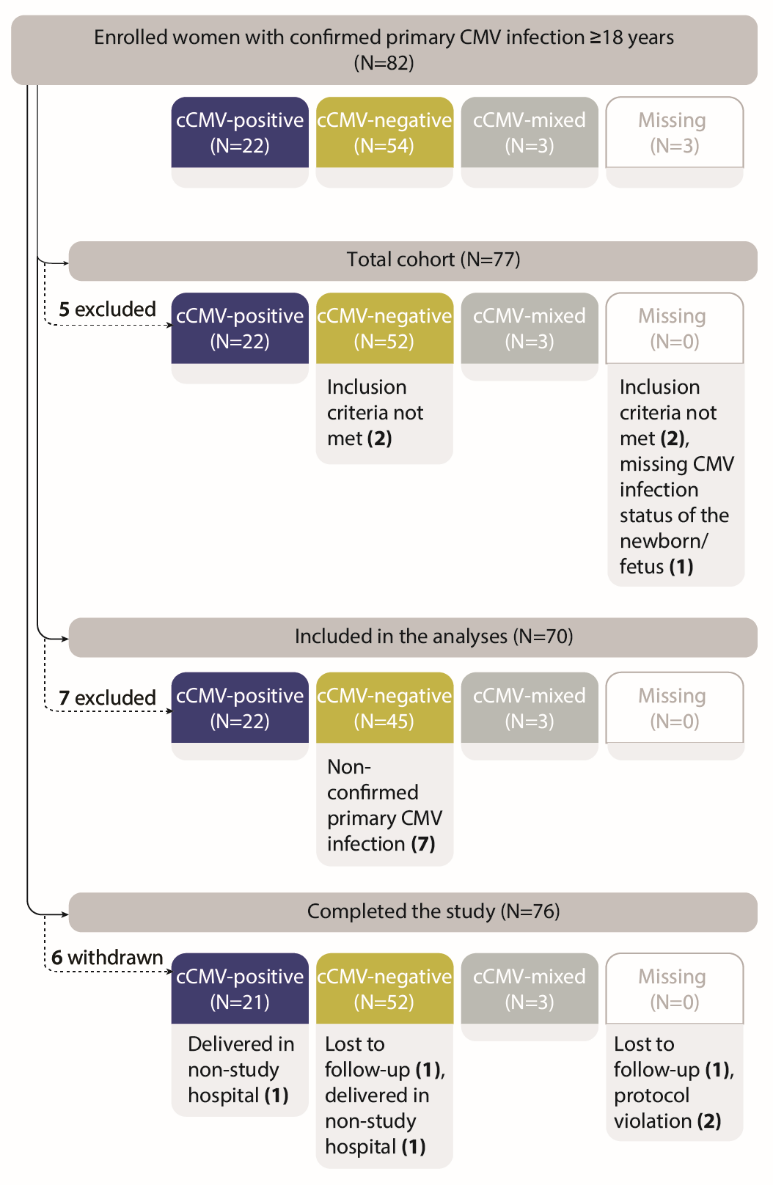


CMV, cytomegalovirus; cCMV, congenital CMV; cCMV-positive, mothers with one/multiple newborn(s)/fetus(es) with cCMV infection; cCMV-negative, mothers with one/multiple newborn(s)/fetus(es) without cCMV infection; cCMV-mixed, mothers with multiple newborns/fetuses with different cCMV infection status; N, number of participants.

Supplementary Figure 3. Anti-CMV antibody titers/concentrations as measured by neutralization assay in the ARPE-19 epithelial cell line (A) and the MRC-5 fibroblast cell line (B), and ELISA assay for anti-pentamer IgG antibodies (C) and anti-gB IgG antibodies (D), by cCMV infection status, sample time since diagnosis, and time of diagnosis (analysis set). Sample time since diagnosis (0–≤4 weeks, >4–≤8 weeks, and >8 weeks) and time of diagnosis (within 12 weeks of pregnancy or later than 12 weeks of pregnancy) are relative to the time point of CMV diagnosis. Box plots (lower quartile [Q1], median, and upper quartile [Q3]) with error bars (minimum and maximum) per group and antibody titers/concentrations for each individual participant are shown.


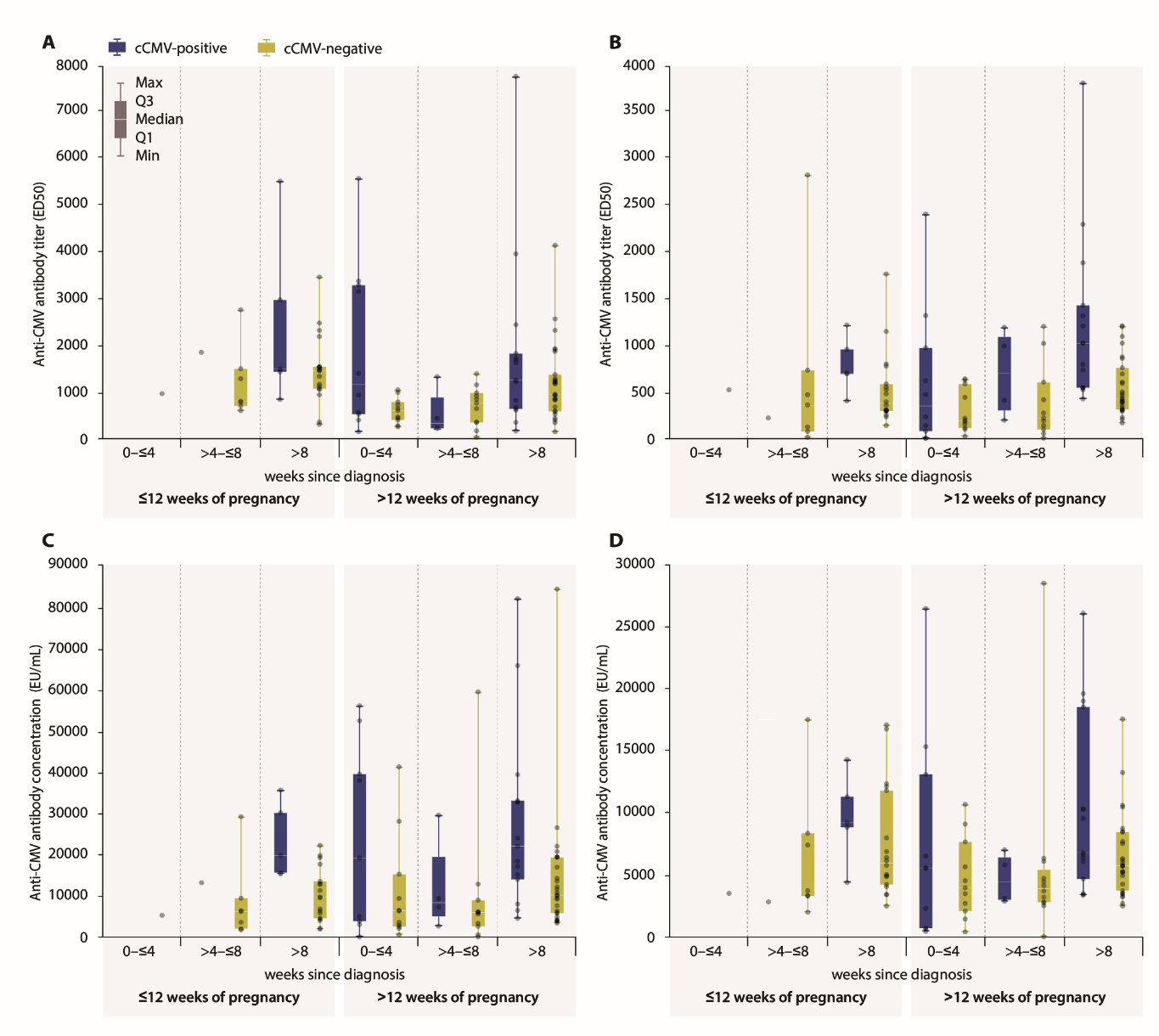


CMV, cytomegalovirus; cCMV, congenital CMV; cCMV-positive, mothers with one/multiple newborn(s)/fetus(es) with cCMV infection; cCMV-negative, mothers with one/multiple newborn(s)/fetus(es) without cCMV infection; ED_50_, endpoint dilution 50%; ELISA, enzyme-linked immunosorbent assay; EU, enzyme-linked immunosorbent assay units; gB, glycoprotein B; IgG, immunoglobulin G.

Supplementary Figure 4. Comparison of viral load values in the cCMV groups, using univariate analysis. Box plots (lower quartile, median, and upper quartile) with error bars (minimum and maximum) are shown to ease the comparison. Values were normalized by taking the logarithm of the original measurements.


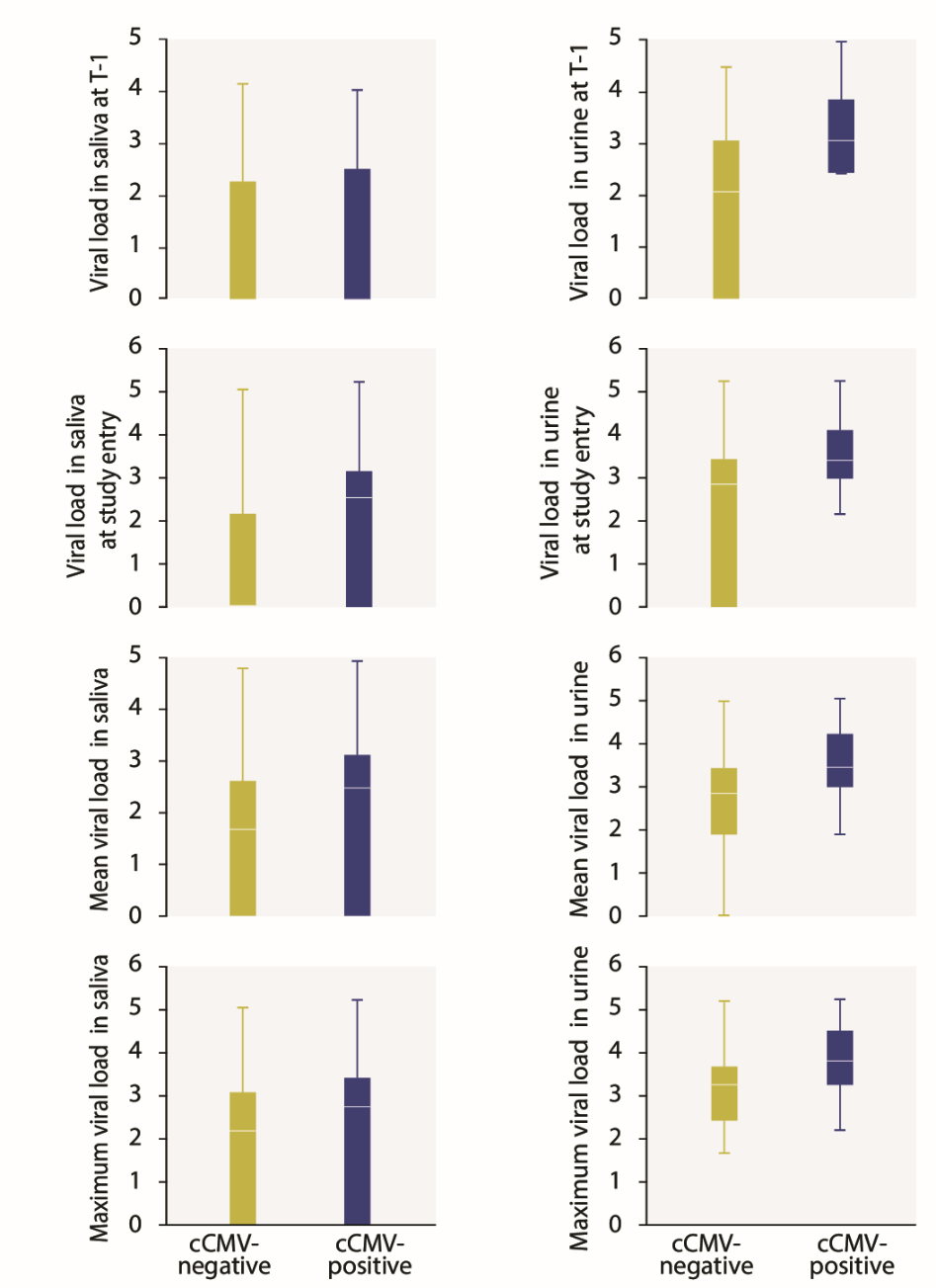


CMV, cytomegalovirus; cCMV, congenital CMV; cCMV-positive, mothers with one/multiple newborn(s)/fetus(es) with cCMV infection (including mothers with multiple newborns/fetuses with different cCMV infection status); cCMV-negative, mothers with one/multiple newborn(s)/fetus(es) without cCMV infection; T-1, last sampling time before pregnancy conclusion.

Supplementary Figure 5. Comparison of viral load values in clusters identified based on dissimilarities, using multivariate analysis. Cluster membership and viral load summary statistics for each type of sample were jointly visualized using UMAP embedding plots. The viral load statistic values reported were normalized by taking the logarithm of the original measurements. Violin plots are plotted to ease the comparison.


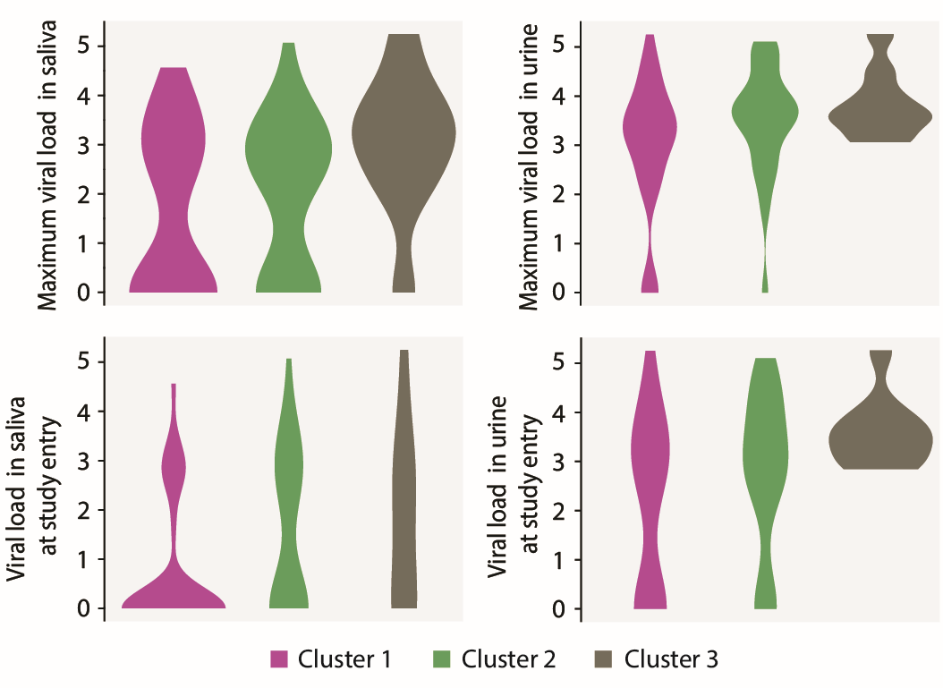


UMAP, uniform manifold approximation projection.

Supplementary Figure 6. Comparison of selected humoral and cell-mediated immune-based biomarker values in the cCMV groups, using univariate analysis, at the last sample collection timepoint before pregnancy. Box plots (lower quartile, median, and upper quartile) with error bars (minimum and maximum) are shown to ease the comparison. Values were normalized by taking the logarithm of the original measurements.


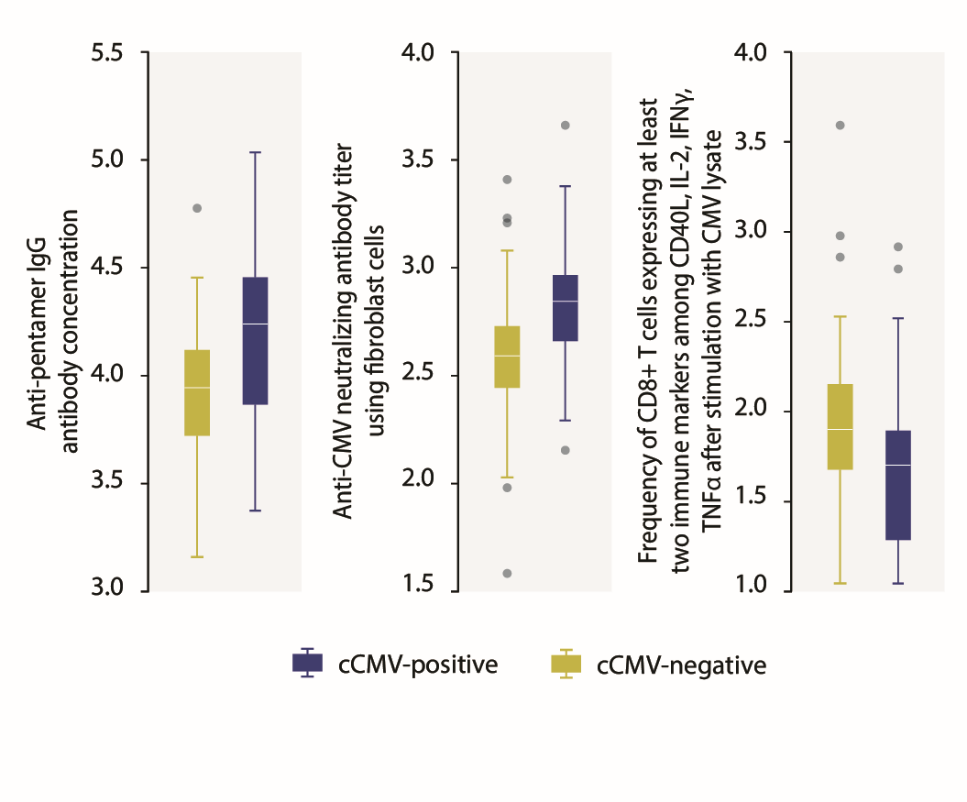


CMV, cytomegalovirus; cCMV, congenital CMV; cCMV-positive, mothers with one/multiple newborn(s)/fetus(es) with cCMV infection (including mothers with multiple newborns/fetuses with different cCMV infection status); cCMV-negative, mothers with one/multiple newborn(s)/fetus(es) without cCMV infection; CD40L, cluster of differentiation 40 ligand; IFNγ, interferon-gamma; IgG, immunoglobulin G; IL-2, interleukin-2; TNFα, tumor necrosis factor-alpha.

## Supplementary Table 1. Descriptive statistics of the anti-gB IgG and anti-CMV tegument protein IgG avidity at study entry and pregnancy conclusion, by cCMV infection status (analysis set)

| **Group** | **Timing** | **Median avidity index (Q1, Q3), %** | **Range (min, max)** |
| --- | --- | --- | --- |
| Anti-gB IgG avidity | |  |  |
| cCMV-positive | Study entry | 22 (18, 29) | 13, 39 |
|  | Pregnancy conclusion | 36 (24, 43) | 19, 51 |
| cCMV-negative | Study entry | 22 (16.5, 36.5) | 11, 64 |
|  | Pregnancy conclusion | 37.5 (29, 43) | 20, 62 |
| cCMV-mixed | Study entry | 22 (14, 28) | 14, 28 |
|  | Pregnancy conclusion | 31 (19, 37) | 19, 37 |
| Anti-CMV tegument protein IgG avidity | | |  |
| cCMV-positive | Study entry | 25 (15, 31) | 9, 46 |
|  | Pregnancy conclusion | 31.5 (24, 42) | 7, 55 |
| cCMV-negative | Study entry | 21.5 (11, 47) | 6, 62 |
|  | Pregnancy conclusion | 28 (18, 44) | 13, 65 |
| cCMV-mixed | Study entry | 22 (22, 22) | 22, 22 |
|  | Pregnancy conclusion | 31 (24, 50) | 24, 50 |

CMV, cytomegalovirus; cCMV, congenital CMV; cCMV-positive, mothers with one/multiple newborn(s)/fetus(es) with cCMV infection; cCMV-negative, mothers with one/multiple newborn(s)/fetus(es) without cCMV infection; cCMV-mixed, mothers with multiple newborns/fetuses with discordant cCMV infection status; gB, glycoprotein B; IgG, immunoglobulin G; Q, interquartile.

## Supplementary Table 2. Adjusted immune responses by visit and cCMV infection status (analysis set)

| Group | Timing | N | Adjusted GMT/C (95% CI) | p-value |
| --- | --- | --- | --- | --- |
| Anti-CMV neutralizing antibodies using epithelial cells | | | | |
| Study entry | cCMV-positive | 21 | 1183.8 (768.2; 1824.2) | 0.0460 |
|  | cCMV-negative | 44 | 690.5 (513.3; 928.9) |  |
| Pregnancy conclusion | cCMV-positive | 19 | 1143.1 (771.2; 1694.2) | 0.8100 |
|  | cCMV- | 42 | 1078.0 (834.1; 1393.2) |  |
| Anti-CMV neutralizing antibodies using fibroblast cells | | | | |
| Study entry | cCMV-positive | 21 | 354.8 (197.5; 637.5) | 0.3875 |
|  | cCMV-negative | 44 | 259.7 (173.8; 388.1) |  |
| Pregnancy conclusion | cCMV-positive | 19 | 983.9 (733.6; 1319.5) | 0.0001 |
|  | cCMV-negative | 42 | 446.2 (368.5; 540.2) |  |
| Anti-pentamer IgG antibodies | | | | |
| Study entry | cCMV-positive | 20 | 13097.3 (7205.7; 23805.9) | 0.0519 |
|  | cCMV-negative | 44 | 6383.8 (4279.1; 9523.8) |  |
| Pregnancy conclusion | cCMV-positive | 19 | 18907.2 (13630.4; 26226.9) | 0.0036 |
|  | cCMV-negative | 39 | 10133.5 (8119.6; 12646.9) |  |
| Anti-gB IgG antibodies | | | | |
| Study entry | cCMV-positive | 21 | 5259.5 (3312.7; 8350.3) | 0.5094 |
|  | cCMV-negative | 45 | 4363.1 (3187.7; 5972.1) |  |
| Pregnancy conclusion | cCMV-positive | 19 | 8903.6 (6864.5; 11548.5) | 0.0125 |
|  | cCMV-negative | 42 | 5884.9 (4967.3; 6972.1) |  |
| Anti-tegument IgG antibodies | | | | |
| Study entry | cCMV-positive | 21 | 6.7 (4.5; 9.8) | 0.0417 |
|  | cCMV-negative | 5 | 4.1 (3.1; 5.3) |  |
| Pregnancy conclusion | cCMV-positive | 19 | 6.5 (4.3; 9.7) | 0.2309 |
|  | cCMV-negative | 42 | 4.8 (3.7; 6.2) |  |
| Specific CD4+ T-cell expressing at least one marker following stimulation with IE1 | | | | |
| Study entry | cCMV-positive | 22 | 134.4 (33.6; 537.0) | 0.5529 |
|  | cCMV-negative | 45 | 80.9 (30.9; 212.3) |  |
| Pregnancy conclusion | cCMV-positive | 19 | 41.3 (8.7; 195.5) | 0.3200 |
|  | cCMV-negative | 42 | 108.1 (39.2; 297. 8) |  |
| Specific CD4+ T-cell expressing at least one marker following stimulation with CMV lysate | | | | |
| Study entry | cCMV-positive | 22 | 1358.1 (428.6; 4303.2) | 0.1744 |
|  | cCMV-negative | 45 | 3588.3 (1608.1; 8007.1) |  |
| Pregnancy conclusion | cCMV-positive | 19 | 897.9 (277.6; 2904.8) | 0.0575 |
|  | cCMV-negative | 42 | 3655.5 (1700.7; 7857.1) |  |
| Specific CD4+ T-cell expressing at least one marker following stimulation with pp65 | | | | |
| Study entry | cCMV-positive | 22 | 156.1 (40.6; 600.4) | 0.7486 |
|  | cCMV-negative | 45 | 203.7 (79.7; 520.1) |  |
| Pregnancy conclusion | cCMV-positive | 19 | 41.2 (7.8; 217.7) | 0.2571 |
|  | cCMV-negative | 42 | 133.5 (45.1; 395) |  |
| Specific CD4+ T-cell expressing at least one marker following stimulation with gB | | | | |
| Study entry | cCMV-positive | 22 | 522.1 (129.3; 2108.9) | 0.8234 |
|  | cCMV-negative | 45 | 632.5 (239.4; 1671.3) |  |
| Pregnancy conclusion | cCMV-positive | 19 | 320.7 (74.5; 1380.3) | 0.1938 |
|  | cCMV-negative | 42 | 1047.7 (404.6; 2712.7) |  |
| Specific CD8+ T-cell expressing at least one marker following stimulation with IE1 | | | | |
| Study entry | cCMV-positive | 22 | 1319.0 (361.2; 4816.3) | 0.3358 |
|  | cCMV-negative | 45 | 2850.1 (1157.2; 7019.7) |  |
| Pregnancy conclusion | cCMV-positive | 19 | 1086.2 (292.8; 4030.2) | 0.0824 |
|  | cCMV-negative | 42 | 4539.0 (1931.3; 10667.9) |  |
| Specific CD8+ T-cell expressing at least one marker following stimulation with CMV lysate | | | | |
| Study entry | cCMV-positive | 22 | 19.8 (5.0; 78.2) | 0.1029 |
|  | cCMV-negative | 45 | 79.8 (30.7; 207.8) |  |
| Pregnancy conclusion | cCMV-positive | 19 | 21.9 (4.8; 99.2) | 0.1298 |
|  | cCMV-negative | 42 | 91.7 (34.3; 245.3) |  |
| Specific CD8+ T-cell expressing at least one marker following stimulation with pp65 | | | | |
| Study entry | cCMV-positive | 22 | 1514.0 (463.8; 4942.0) | 0.9057 |
|  | cCMV-negative | 45 | 1388.8 (609.6; 3163.8) |  |
| Pregnancy conclusion | cCMV-positive | 19 | 1175.5 (377.3; 3662.8) | 0.4697 |
|  | cCMV-negative | 42 | 1958.1 (933.5; 4107.0) |  |
| Specific CD8+ T-cell expressing at least one marker following stimulation with gB | | | | |
| Study entry | cCMV-positive | 22 | 268.2 (70.6; 1019.9) | 0.9512 |
|  | cCMV-negative | 45 | 255.1 (100.7; 646.2) |  |
| Pregnancy conclusion | cCMV-positive | 19 | 168.7 (41.4; 687.5) | 0.4237 |
|  | cCMV-negative | 42 | 339.2 (135.8; 847.4) |  |

CI, confidence interval; CMV, cytomegalovirus; cCMV, congenital CMV; cCMV-positive, mothers with one/multiple newborn(s)/fetus(es) with cCMV infection; cCMV-negative, mothers with one/multiple newborn(s)/fetus(es) without cCMV infection; gB, glycoprotein B; GMT/C, geometric mean titer/concentration; IE1, immediate-early 1 protein; IgG, immunoglobulin G; N, total number of samples; pp65, phosphoprotein 65.

Notes: Data were not available for all women at all time points due to differences in gestational age at study entry and/or due to attendance.

Adjusted GMT/Cs and p-values were calculated using a general linear model with cCMV serostatus as a fixed factor and time of diagnosis (≤12 weeks or >12 weeks of pregnancy) and sample time since diagnosis (0–≤4 weeks, >4–≤8 weeks, or >8 weeks) as covariates.

**Supplementary Table 3. Wilcoxon Rank-sum statistics and corresponding p-values for viral load-based statistics.** The null hypothesis was: no difference between cCMV-positive and cCMV-negative groups. P-values<0.05 are presented in bold. Specific p-values should not be interpreted within the context of statistical significance.

| Statistic | Wilcoxon rank-sum statistic | p-value |
| --- | --- | --- |
| Urine samples |  |  |
| Mean viral load | 310.5 | **0.002** |
| Maximum viral load | 349.5 | **0.009** |
| Viral load at study entry | 371.0 | **0.018** |
| Viral load at last sampling before pregnancy conclusion | 350.5 | **0.008** |
| Saliva samples |  |  |
| Mean viral load | 421.0 | 0.074 |
| Maximum viral load | 425.5 | 0.084 |
| Viral load at study entry | 373.0 | **0.008** |
| Viral load at last sampling before pregnancy conclusion | 467.0 | 0.169 |

cCMV, congenital cytomegalovirus; cCMV-positive, mothers with one/multiple newborn(s)/fetus(es) with cCMV infection (including mothers with multiple newborns/fetuses with different cCMV infection status); cCMV-negative, mothers with one/multiple newborn(s)/fetus(es) without cCMV infection.

**Supplementary Table 4. Multivariate logistic regression model for humoral and CMI-based immunological markers.** Analyses were performed separately for each set of biomarkers (humoral and CMI-based). P-values<0.05 are presented in bold. However, all analyses were exploratory, and p-values should not be interpreted strictly in terms of statistical significance.

| Variable | Coefficient estimate | Standard error | z-stat | p-value |
| --- | --- | --- | --- | --- |
| Humoral immunological markers |  |  |  |  |
| Intercept | -3.291 | 4.351 | -0.757 | 0.450 |
| Anti-pentamer IgG antibody concentration | 2.725 | 1.232 | 2.211 | **0.027** |
| Anti-tegument IgG antibody concentration | 1.993 | 1.009 | 1.975 | **0.048** |
| Anti-gB IgG antibody concentration | -2.720 | 1.401 | -1.941 | 0.052 |
| Anti-CMV neutralizing antibody titer using fibroblast cells | 1.429 | 1.042 | 1.371 | 0.170 |
| Anti-CMV neutralizing antibodies titer using epithelial cells | -1.114 | 1.047 | -1.064 | 0.287 |
| CMI-based immunological markers: frequency of T cells expressing at least two immune markers among CD40L, IL-2, IFNγ, TNFα after stimulation with CMV antigens | | | | |
| Intercept | -8.85 | 4.47 | -1.982 | **0.0474** |
| CD4+, stimulation with IE1 | 2.82 | 1.34 | 2.095 | **0.0362** |
| CD4+, stimulation with CMV lysate | 0.69 | 0.93 | 0.745 | 0.4563 |
| CD4+, stimulation with pp65 | -1.15 | 1.18 | -0.970 | 0.3322 |
| CD4+, stimulation with gB | 0.74 | 0.52 | 1.426 | 0.1538 |
| CD8+, stimulation with IE1 | -0.27 | 0.47 | -0.572 | 0.5674 |
| CD8+, stimulation with CMV lysate | -1.59 | 0.66 | -2.398 | **0.0165** |
| CD8+, stimulation with pp65 | 0.96 | 0.59 | 1.636 | 0.1019 |
| CD8+, stimulation with gB | 0.27 | 0.51 | 0.524 | 0.6000 |

cCMV, congenital cytomegalovirus; CD40L, cluster of differentiation 40 ligand; CMI, cell-mediated immunity; gB, glycoprotein B; IE1, immediate-early 1 protein; IFNγ, interferon-gamma; IgG, immunoglobulin G; IL-2, interleukin-2; pp65, phosphoprotein 65; TNFα, tumor necrosis factor-alpha.
